# The Nimrod transmembrane receptor Eater is required for hemocyte attachment to the sessile compartment in *Drosophila melanogaster*

**DOI:** 10.1242/bio.201410595

**Published:** 2015-02-13

**Authors:** Andrew J. Bretscher, Viktor Honti, Olivier Binggeli, Olivier Burri, Mickael Poidevin, Éva Kurucz, János Zsámboki, István Andó, Bruno Lemaitre

**Affiliations:** 1 Global Health Institute, School of Life Sciences, Ecole Polytechnique Fédérale de Lausanne (EPFL), Station 19, 1015 Lausanne, Switzerland; 2 Institute of Genetics Biological Research Centre of the Hungarian Academy of Sciences, P.O. Box 521, Szeged H-6701, Hungary; 3 Bioimaging and Optics Platform, School of Life Sciences, Ecole Polytechnique Fédérale de Lausanne (EPFL), Station 19, 1015 Lausanne, Switzerland; 4 Centre de Génétique Moléculaire, CNRS/Université Pierre et Marie Curie, 91198 Gif-sur-Yvette, France

**Keywords:** EGF-like, Eater, Hemocyte, Receptor, Sessile

## Abstract

Eater is an EGF-like repeat transmembrane receptor of the Nimrod family and is expressed in *Drosophila* hemocytes. Eater was initially identified for its role in phagocytosis of both Gram-positive and Gram-negative bacteria. We have deleted *eater* and show that it appears to be required for efficient phagocytosis of Gram-positive but not Gram-negative bacteria. However, the most striking phenotype of *eater* deficient larvae is the near absence of sessile hemocytes, both plasmatocyte and crystal cell types. The *eater* deletion is the first loss of function mutation identified that causes absence of the sessile hemocyte state. Our study shows that Eater is required cell-autonomously in plasmatocytes for sessility. However, the presence of crystal cells in the sessile compartment requires Eater in plasmatocytes. We also show that *eater* deficient hemocytes exhibit a cell adhesion defect. Collectively, our data uncovers a new requirement of Eater in enabling hemocyte attachment at the sessile compartment and points to a possible role of Nimrod family members in hemocyte adhesion.

## INTRODUCTION

Circulating immune cells express many cell surface receptors, following their specialized role in host defense. These roles include cell adhesion, cell-cell recognition, phagocytosis, chemokine-binding and others (Alberts et al., 2002). In *Drosophila* and other insects, hemocytes are circulating immune cells, which participate in the humoral and cellular immune defense reactions against microbes and parasites (Lemaitre and Hoffmann, 2007; Honti et al., 2014). *Drosophila* hemocytes express many surface receptors, some of whose functions are not well understood or have not been studied (Kurucz et al., 2003; Kurucz et al., 2007; Somogyi et al., 2008; Ulvila et al., 2011). Here, we re-visit the function of Eater, an EGF-like repeat Nimrod receptor that is specifically expressed in *Drosophila* hemocytes (Kocks et al., 2005; Kurucz et al., 2007).

*Drosophila* larvae have two types of hemocytes in the unchallenged state: plasmatocytes, which are macrophage-like, and crystal cells, rounded hemocytes which contain crystals of pro-phenoloxidases, the enzyme zymogen of phenoloxidase that catalyses the melanization reaction against parasites or septic injury (Rizki et al., 1980; Rizki and Rizki, 1992; Lanot et al., 2001). Larval hemocytes are found in three compartments: (i) the lymph glands that function as a reservoir releasing hemocytes after parasitic infection, (ii) in the circulation and, (iii) in the sessile patches (Lanot et al., 2001; Evans and Banerjee, 2003; Jung et al., 2005; Crozatier and Meister, 2007; Honti et al., 2010; Makhijani et al., 2011; Makhijani and Brückner, 2012). Sessile hemocytes are attached to the internal surface of the larval body wall, forming patches, some of which are closely associated with secretory cells called oenocytes, as well as the endings of peripheral neurons (Makhijani et al., 2011; Makki et al., 2014). Hemocytes continuously exchange between sessile patches and the circulation (Babcock et al., 2008; Welman et al., 2010). Interestingly, hemocytes leave the sessile patches and enter the circulation upon wasp infestation or mechanical stimulation of the cuticle by brushing (Márkus et al., 2009; Makhijani et al., 2011). The formation and function of sessile hemocyte patches is not yet established but it has been proposed that they form a diffuse hematopoietic organ (Márkus et al., 2009; Makhijani et al., 2011).

Eater is an EGF-like repeat single pass transmembrane receptor of the Nimrod family (Kocks et al., 2005; Kurucz et al., 2007). Eater has 32 EGF-like or Nim repeats in the extracellular domain, a transmembrane domain and cytoplasmic tail with no identified functional domains (Kurucz et al., 2007). *eater* was initially identified as a plasmatocyte receptor encoding gene required for efficient phagocytosis of *S. aureus* and *E. coli* in *Drosophila* (Kocks et al., 2005). The contribution of *eater* to phagocytosis of different bacterial types was measured in S2 cells expressing an RNAi knock down of *eater* and in *ex vivo* hemocytes of larvae carrying overlapping deficiencies ablating *eater* and seven flanking genes (Kocks et al., 2005). Additionally, it has been shown that a recombinant fragment of the Eater extracellular domain can bind to bacteria or bacterial products and that Eater is cell-surface expressed (Chung and Kocks, 2011).

Here we have generated a knockout of *eater* by homologous recombination and showed its requirement for efficient phagocytosis of Gram-positive and but not Gram-negative bacteria. Larvae lacking *eater* have more than two times the number of circulating hemocytes compared to wild type controls. Imaging the sessile compartment reveals that *eater* deficient larvae lack nearly all sessile hemocytes, both plasmatocyte and crystal cell types. We show that Eater is required cell-autonomously in individual plasmatocytes for their presence at the sessile compartment. Allowing hemocytes to adhere to a glass slide reveals that *eater* deficient hemocytes exhibit a cell adhesion defect. Collectively, our data uncovers a new requirement for the transmembrane receptor Eater in the formation of the hemocyte sessile compartment.

## MATERIALS AND METHODS

### *Drosophila* stocks and methodology

Wild type *Oregon^R^* flies and *w^1118^(BL5905)* were used as controls, unless otherwise indicated. Fly larvae were reared at a density of 30 female flies with 15 males per large vial laying for 24 hrs. We generated and used stocks *w^1118^;; eater^1^*, *w^1118^; HmlΔGAL-4, UASGFP* and *w^1118^; HmlΔGAL-4, UASGFP; eater^1^*, *w^1118^;; Df(3R)6206/TM6c* (derived from BL7685) and *w^1118^;; Df(3R)791/TM6c* (derived from BL27363), *yw, lzGAL4, UAS-GFP* and *yw, lzGAL4, UAS-GFP;; eater^1^, Bc(II)* and *Bc(II); eater^1^, w^1118^; HmlΔGAL-4, UASGFP; UAS-eater.RNAi/TM3, actGFP, Ser* and *w^1118^;; UAS-eater.RNAi/TM3,actGFP,Ser, yw, lzGAL4, UAS-GFP; HmlΔDsRed.nls, w^1118^, EaterGAL4, UAS-2xeYFP; BcF6-CFP (P1^+^); msn9-mCherry* and *w, EaterGAL4, UAS-2xeYFP; BcF6-CFP (P1^+^); msn9-mCherry, eater^1^*. The *HmlΔGAL-4* transgene drives expression in plasmatocytes only (Sinenko et al., 2004; Makhijani et al., 2011). The *UAS-eater.RNAi* flies were derived from stock *6124R-2* of the National Institute of Genetics (NIG), Japan. Experiments were repeated at least twice on 2 separate days. Unless otherwise indicated, data was analysed in Excel 2011 (Microsoft) and Prism v5.0a (Graphpad) and significance tests performed using Students *t* test. For Fig. 2E, statistical analyses were performed using the R program (R Development Core Team, 2008) with the R commander graphical interface (Fox, 2005).

**Fig. 1.**
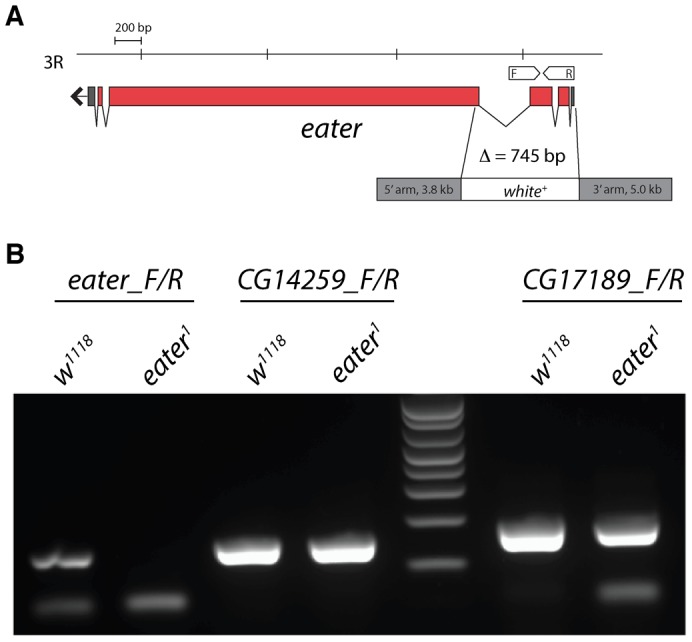
Gene targeting and deletion of *eater*. (A) Deletion of *eater* by homologous recombination. The *eater* gene is on the R arm of chromosome 3 and encodes a single transcript. Exons are represented by red boxes, introns by adjoining lines and 5′ and 3′ UTRs by grey boxes. Eye colour was transformed from white to red eye by the *white^+^* marker. F, forwards and R, reverse primers were used in RT-PCR (B). (B) RT-PCRs confirming functional deletion of *eater* but not flanking genes *CG14259* and *CG17189*. In the *eater_F/R* reaction, the expected 122 bp RT-PCR product is present in *w^1118^* and absent in the *eater^1^* mutant. In *CG14259_F/R* and *CG17189_F/R* reactions, the expected 153 bp and 178 bp products, respectively, are present in both *w^1118^* and the *eater^1^* mutant.

**Fig. 2.**
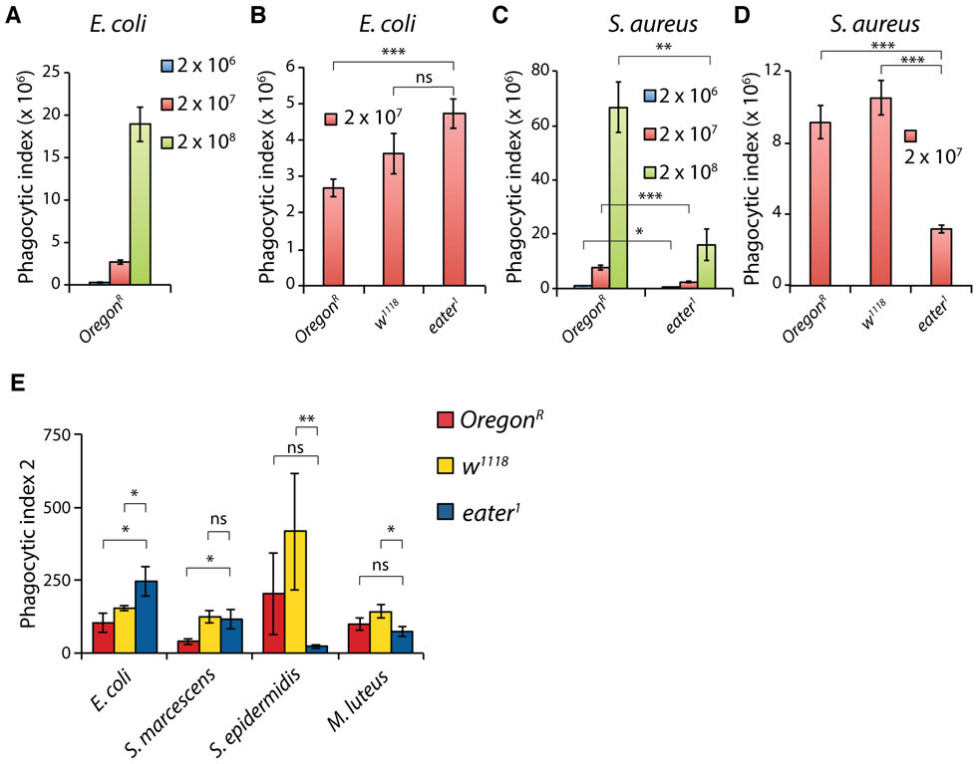
*eater^1^* null hemocytes have defects in phagocytosis of Gram-positive but not Gram-negative bacteria. (A,B) Phagocytosis of AF488-labelled heat-killed *Escherichia coli* by *ex vivo* hemocytes from *Oregon^R^*, *w^1118^* and *eater^1^* mutant larvae. Larvae were bled, hemocytes collected, mixed with heat-killed bacteria and incubated at room temperature for 20 min. (A) Phagocytosis index of *Oregon^R^* hemocytes incubated with 2×10^6^, 2×10^7^ or 2×10^8^ *E. coli* (n = 6, n = 12 and n = 2, respectively). (B) Phagocytosis by *eater^1^* mutant hemocytes when incubated with 2×10^7^ *E. coli* is like wild type (n = 6 or more, each). In this and all other figures, unless otherwise indicated, statistical tests were performed using Students *t* test, *p<0.05, **p<0.01 and ***p<0.001 and ‘ns’ indicates not significant. Error bars represent SEM. (C,D) Phagocytosis of AF488-labelled heat-killed *Staphylococcus aureus* by *ex vivo* hemocytes from *Oregon^R^*, *w^1118^* and *eater^1^* mutant larvae. (C) *eater^1^* null hemocytes are inefficient at phagocytosing *S. aureus* compared to wild type hemocytes, at all titres tested, 2×10^6^, 2×10^7^ and 2×10^8^ *S. aureus* (n = 5 or more, each). Performed using 20 larval bleeds per assay, as in A,B. (D) The phagocytic index of hemocytes incubated with 2×10^7^ *S. aureus* (n = 6 or more, each). In this latter plot, equal numbers of hemocytes were used across genotypes by compensating for variation in the number of hemocytes per larva (see Fig. 3A; Materials and methods) by varying the number of larvae bled. (E) Phagocytosis of AF488-labelled heat-killed Gram-negative *Escherichia coli* and *Serratia marcescens* and Gram-positive *Staphylococcus epidermidis* and *Micrococcus Luteus* by Oregon^R^, *w^1118^* and *eater^1^ ex vivo* larval hemocytes. A titre of 2×10^6^ bacteria was used. *n* number of experimental repeats; *E.c.*; Oregon (6), *w^1118^* (3) and *eater^1^* (5); *S.m.*; Oregon (4), *w^1118^* (3) and *eater^1^* (5); *S.e.*; Oregon (4), *w^1118^* (4) and *eater^1^* (5); *M.l*. Oregon (6), *w^1118^* (5) and *eater^1^* (5). Mean Phagocytic indices are plotted using the same formula as in A,B but different arbitrary fluorescence units. For each condition we performed a Shapiro-Wilk test of normality. For non-parametric datasets, we performed pair-wise Wilcoxon tests.

### Gene targeting of *eater*

Deletion limits of the *eater^1^* allele: 5′-GTTGTATACTTAAAGACACC…[insert]… GGGATGTAGTCGAGGAACCT-3′. The 5′ and 3′ homology arms, 5.0 kb and 3.8 kb, respectively, were PCR amplified from BACR21O10 clone (CHORI) using Hot-start PHusion Polymerase (New England Biolabs). The 5′ arm was inserted between *Not*I and *Nhe*I sites, and the 3′ arm was inserted between *Spe*I and *Asc*I sites of the gene targeting vector piHR (Baena-Lopez et al., 2013). A donor transgenic stock, *w^1118^; eater_piHR1 (II)*, was generated by transformation (Fly Facility, France) of starting stock *w^1118^(BL5905)* and used for *hsFLP* and *hs*-I-*Sce*I mediated gene targeting (Baena-Lopez et al., 2013). Using this method, we recorded a knockout efficiency of ∼1/5000 of the F_2_ progeny were bonafide *eater* knockouts.

### *Ex vivo* larval hemocyte phagocytosis assay

**Fig. 3.**
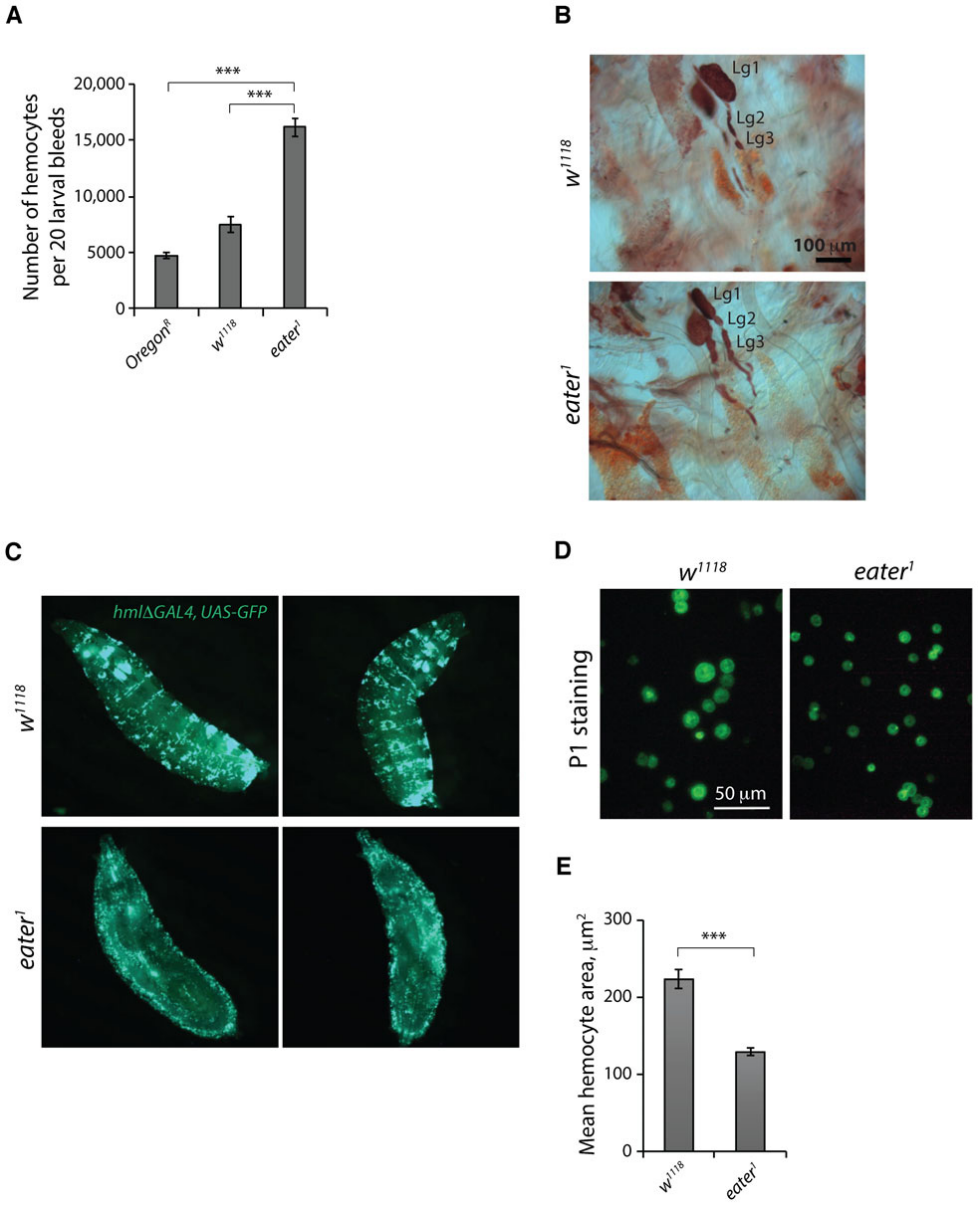
*eater* is required in plasmatocytes for binding of sub-epidermal body wall tissue. (A) Aggregated flow cytometry measurements from phagocytosis assays (Fig. 2A,B) of the number of hemocytes per 20 larval bleeds of Oregon R, *w^1118^* and *eater^1^* mutant larvae. Oregon^R^, n = 41; *w^1118^*, n = 12; e*ater^1^* mutant, n = 37. (B) Lymph glands of *w^1118^* and *eater^1^* mutant third instar larvae dissected and stained *in situ* with anti-Hemese primary antibody and HRP-conjugated secondary antibody, developed with the chromogen AEC. Lymph gland primary, secondary and tertiary lobes are labeled Lg1, Lg2 and Lg3, respectively. (C) Live mount images of *w^1118^* and *eater^1^* third instar larvae expressing *UAS-GFP* in plasmatocytes driven by the *HmlΔGAL4* transgene. (D) Fixed hemocytes from *w^1118^* and *eater^1^* larvae spread on HCl-cleaned glass slides, stained with the plasmatocyte-specific P1 antibody. (E) Mean cell areas of hemocytes from *w^1118^* and *eater^1^* larvae spread on HCl-cleaned glass slides.

We combined several existing protocols (Kocks et al., 2005; Watson et al., 2005; Kurucz et al., 2007) to measure phagocytosis by larval hemocytes. For full details, see Neyen and colleagues (Neyen et al., 2014). Briefly, phagocytosis of fluorescent heat-killed bacteria was quantified using a flow cytometer (BD Accuri, USA) to measure both the fraction of cells phagocytosing and the intensity of phagocytosis. Wandering third instar larvae were bled in cold Schneiders medium (Gibco) containing 1 nM phenylthiourea (PTU, Sigma). Hemocytes were incubated in 100 µl volumes in ultra low attachment 96-well plates (Costar no. 3474, Corning) at room temperature (RT) for 10 min. Then, 10 µl of a homogeneous suspension of Alexa-Fluor AF488 heat-killed bacteria (Molecular Probes) of titre 2×10^6^, 2×10^7^ or 2×10^8^ in Schneiders/PTU was added and the plate incubated at RT for 20 min. After incubation, the fluorescence of extracellular bacterial particles was quenched by adding trypan blue (Sigma). The fluorescence intensity of single hemocytes, not part of multicellular hemocyte clusters, was measured on red and green fluorescence channels with a 488 nm laser and BP530/30 and BP585/40 band-pass filters, respectively. The mean fluorescence intensity of a hemocyte population without bacteria added was used to define the gate for the phagocytosing hemocyte population. The phagocytic index was calculated as follows:
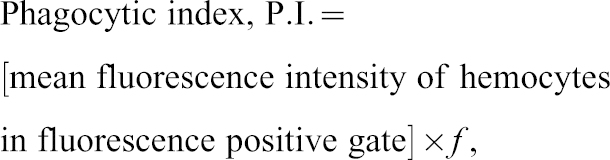
where *f* is equal to the fraction of hemocytes phagocytosing:
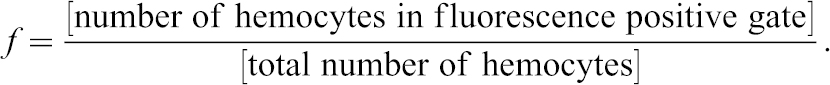


Note, the number of circulating hemocytes per larva can vary dramatically between genotypes. On average, 20 Oregon^R^ larval bleeds yield ∼5000 hemocytes, 20 *w^1118^ (BL5905)* larval bleeds yield ∼7,500 hemocytes and 20 *w^1118^;; eater^1^* larval bleeds yield ∼16,000 hemocytes (Fig. 3A). Therefore to achieve cell-matched assays across genotypes, we adjusted the number of larval bleeds between genotypes. Therefore, we used 13 *w^1118^ (BL5905)* larval bleeds and 6 *w^1118^;; eater^1^* larval bleeds per assay to achieve 20 Oregon^R^ larval bleed-equivalents across all genotypes.

### Live imaging of third instar larvae

For whole larval imaging, cleaned third instar larvae were mounted in cold PBS between two glass slides. Images were captured on a Leica MZ-16F fluorescence microscope with Leica Application Suite version 2.8.1. For live imaging of sessile patches, larvae were mounted dorsal side up on a 2% agarose pad on a glass slide atop a 9 cm petri plate filled with ice. Dermabond glue (Ethicon, US) was applied along the length of the larva and allowed to set. The glued animal was immersed in PBS and a cover glass placed dorsally. All imaging was completed within 20 min. Images of sessile patches were captured with 10× or 20× objectives, a CCD B/W camera (detector size 6.45 µm) mounted on a Zeiss AxioImager Z.1 and Axiovision software (Carl Zeiss).

For confocal imaging, live samples were inverted and mounted on an inverted Olympus IX 81 microscope with confocal scanner unit (CSU-W1, Yokogawa, Japan) and imaged with a ×60/NA 1.42 oil U PLAN S APO objective. Images were captured on an EMCCD ImagEM B/W camera (detector size 16 µm, Hamamatsu, Japan) and analysed in VisiView (Visitron Systems, Germany) and Fiji (ImageJ). Z-stacks were typically 25–50 optical slices deep with a slice separation of 0.5 µm.

### Generation of MARCM clones

MARCM GFP+ hemocyte clones were generated by embryonic heat shock induction (38°C, 1 h) of the *hsFLP* gene as described in previously (Minakhina et al., 2007). To generate MARCM clones, we used *y, hsFLP, ptubGAL4, UAS-GFP;; FRT82B, ptubGAL80/TM6B, yw;; FRT82B *and *yw;; FRT82B, eater^1^*.

### Lymph gland and hemocyte immunostaining

Lymph glands from synchronized early third instar larvae were dissected following standard protocols (Evans et al., 2014). Lymph glands were stained with mouse anti-Hemese primary antibody and horseradish peroxidase HRP-conjugated anti-mouse Ig and AEC as a developer chromogen. Hemocyte immunostaining was performed as previously described (Márkus et al., 2009), except that hemocytes were allowed to adhere in Schneiders medium supplemented with 5% fetal bovine serum (FBS) to HCl-cleaned glass slides at 25°C for 3 h. Glass slides were washed in detergent and water, washed extensively with running tap water, incubated in 1 M HCl overnight, re-rinsed extensively with running tap water, rinsed in distilled water, rinsed in 70% ethanol and dried at 37°C.

### Hemocyte cell area measurement

Spread hemocytes were prepared as for immunostaining, except that cells were stained with AF488-phalloidin (Molecular Probes) and mounted in Vectashield-DAPI (Vector labs). Mosaic 2×2 images of hemocytes were captured with a ×20 objective on GFP and DAPI channels using Zeiss Axiovision software. Individual image tiles of mosaic images were extracted using an ImageJ macro (‘extract_czi.ijm’ file). The extracted images were loaded into a CellProfiler (www.cellprofiler.org) pipeline to segment cells and extract cell areas. First, cell nuclei were detected using data from the DAPI channel, then cell area limits were detected by expanding the cell nuclei to the edges of the GFP signal. Cell areas were computed from these segmentations (‘Cell_Profiler_Analysis.project’ file). Both these files are available upon request.

### Crystal cell counting methods

For crystal cell visualization by heating, ten third instar larvae were heated in 0.5 ml PBS in eppendorf tubes for 30 min at 67°C. Larvae were recovered, mounted between two glass slides over a white background and imaged. For quantification, black puncta were counted circumferentially in the posteriormost segments A6, A7 and A8.

To count live crystal cells, wandering third instar larvae, five at a time, carrying the *BcF6-CFP* label were selected, washed and vortexed for 1 min at max speed to release sessile crystal cells. Larval hemocytes were dissected to 5.5 µl of Schneiders medium containing 1 nM phenylthiourea (PTU, dissolved in DMSO) in 10 mm diameter wells of 8-well glass slides (silane surface, Teflon mask, Tekdon Inc., Florida). This volume is sufficient to fill the well when a coverslip (12 mm diameter, Menzel Glåser) is placed over the well. Five larvae were dissected per well. Circular mosaic images (13 columns × 17 rows) of the entire well under CFP illumination were immediately captured and the number of CFP-expressing crystal cells counted directly by eye from the mosaic image. To count black cells, *Bc*-carrying larvae were treated similarly but 20 larvae were vortexed and hemocytes dissected to 120 µl of Schneiders medium, 1 nM PTU. The resulting cell suspension was mounted over a 1 mm^2^ grid hemocytometer (Preciss, France), the number of black cells counted and the number of black cells per larva derived.

### Live imaging of crystal cell rupture

Two third instar larvae were dissected in 6 µl PBS-0.1% BSA on glass slides (Menzel-Glaser Superfrost). Immediately a 12 mm diameter cover glass (Menzel-Glaser) was placed and sample mounted. Crystal cells were located under CFP illumination and imaged with a ×100 oil objective under DIC III on a Zeiss AxioImager Z.1. Time between dissection and imaging was typically less than 2 minutes.

## RESULTS

### Deletion of the *eater* gene by homologous recombination

To investigate Eater function, we deleted *eater* using an optimized method of gene-targeting (Baena-Lopez et al., 2013). Gene targeting in the *w^1118^* genetic background yielded *eater^1^*, a 745 bp deletion removing the ATG translation start site, the first and second exons, 18 bp of the third exon together with insertion of a 7.9 kb cassette carrying the *white^+^* gene (Fig. 1A; Materials and Methods). We confirmed functional deletion of *eater* and integrity of flanking genes by RT-PCR from total RNA (Fig. 1B). Flies carrying the *eater^1^* lesion appear developmentally wild type, consistent with previous findings that plasmatocyte-deficient flies are mostly viable (Charroux and Royet, 2009; Defaye et al., 2009).

### Eater appears to be required for efficient phagocytosis of Gram-positive but not Gram-negative bacteria

We first used the *eater^1^* deletion to ask whether *eater* is required for phagocytosis of heat-killed, labeled bacteria, as previously reported (Kocks et al., 2005). We employed an *ex vivo* phagocytosis assay in which larval hemocytes were incubated with 2×10^6^, 2×10^7^ or 2×10^8^ Alexa Fluor 488-labeled heat-killed bacteria and run on a flow cytometer to measure the fluorescence of hemocytes. To quantify phagocytosis, we used a phagocytic index (P.I.) equal to the fraction of cells phagocytosing multiplied by the mean fluorescence intensity of the phagocytosing cell population. As controls, we used hemocytes from *w^1118^* larvae, carrying the same genetic background as *eater^1^* mutant larvae, and hemocytes from wild type *Oregon^R^* larvae. We observed that phagocytosis of Gram-negative *E. coli* by *eater^1^* deficient hemocytes was similar or even more than that of control *Oregon^R^* wild type hemocytes (Fig. 2A,B). Contrastingly, phagocytosis of Gram-positive *Staphylococcus aureus* was defective in *eater^1^* null hemocytes compared to wild-type controls at all titres tested (Fig. 2C,D), consistent with previous analyses (Kocks et al., 2005). We extended our analysis to other Gram-positive and Gram-negative bacteria. *eater^1^*null hemocytes phagocytosed the Gram-negative *Serratia marcescens* to wild type levels, but were deficient in phagocytosis of the Gram-positive *Staphylococcus epidermidis* and *Micrococcus luteus* (Fig. 2E). Together these data indicate that phagocytosis of the Gram-positive bacteria *S. aureus*, *S. epidermidis* and *M. luteus*, but not the Gram-negative bacteria *E. coli* and *S. marcescens* by plasmatocytes, the major macrophage-like cell type in *Drosophila*, is defective in *eater^1^* null larvae.

### Sessile plasmatocytes are absent or almost absent in *eater^1^* null larvae

While examining phagocytosis, we noticed that dissected *eater^1^* null larvae release more than three times the number of hemocytes that wild type *Oregon^R^* larvae release and more than two times more than *w^1118^* larvae (Fig. 3A). The high number of circulating hemocytes prompted us to investigate the anatomy of the hemocyte compartments in third instar *eater^1^* larvae. In wandering third instar larvae, around one third of all hemocytes are present in the lymph glands, one third are circulating and one third are sessile (Lanot et al., 2001; Jung et al., 2005; Crozatier and Meister, 2007). The lymph gland does not normally release hemocytes except upon wounding or immune challenge by parasitoids or at metamorphosis (Lanot et al., 2001; Honti et al., 2010). We first asked whether a defect in lymph gland organization could explain the higher number of circulating hemocytes in *eater^1^* larvae. Dissecting the lymph glands, we observed that those of *eater^1^* larvae were not visibly different in size to those of *w^1118^* control larvae (Fig. 3B).

Recent studies show that hemocytes exchange between a circulating state and a static or sessile state in which they are body wall-bound (Babcock et al., 2008; Welman et al., 2010; Makhijani et al., 2011). The sessile hemocyte compartment is visible as a striped pattern of hemocyte patches along the length of the larva (Zettervall et al., 2004) and comprises plasmatocytes and crystal cells (Lanot et al., 2001). To explore hemocyte pattern in the absence of Eater, we combined the plasmatocyte reporter *HmlΔGAL4, UAS-GFP* with the *eater^1^* mutation and imaged whole larvae. The sessile plasmatocyte striped pattern evident in *w^1118^* larvae was absent in *eater^1^* mutant larvae (Fig. 3C). All or almost all plasmatocytes in *eater^1^* larvae are in circulation and appear not to enter the sessile state. This ‘no sessile plasmatocyte’ phenotype was phenocopied in trans-heterozygous larvae carrying *eater^1^* over the deficiencies *Df(3R)6206* or *Df(3R)791* (data not shown), suggesting that absence of sessile hemocytes was indeed caused by the lesion in the *eater* gene. Together these data suggest that Eater is required for plasmatocytes to enter the sessile state, and that consequently, *eater* deficient larvae have close to double the number of freely circulating plasmatocytes that wild type larvae have.

### *eater* is required cell-autonomously for plasmatocytes to enter the sessile state

Eater could either be required in plasmatocytes for them to enter the sessile state or in a different cell type. To address this, we knocked down *eater* transcripts in plasmatocytes using *HmlΔGAL4* combined with a *UAS-eater RNAi*. Knocking down *eater* in the *Hml* positive lineage alone was sufficient to cause a near absence of sessile plasmatocytes (see Fig. 5B). Next we used a clonal analysis to ask whether *eater* is required in individual plasmatocytes for them to become sessile. We generated *gfp* positive clones of *eater^1^* mutant hemocytes in mosaic larvae that were otherwise *eater^1^/+* heterozygous using MARCM (mosaic analysis with a repressible cell marker) (Lee and Luo, 1999). To image plasmatocytes *in vivo*, we immobilized third instar larvae to agar pads by gluing (see Materials and Methods). Imaging control *gfp* wild type hemocyte clones in third instar larvae showed that as the glued larva moves, many sessile plasmatocytes keep the same position within the elapsed time (supplementary material Fig. S1 and Movie 1). In contrast, imaging *gfp* positive *eater^1^* mutant plasmatocytes showed that the majority of *gfp+* hemocytes do not remain stationary with respect to the cuticle as the larva moved, indicating that the majority of *eater* deficient hemocytes lacked sessility (supplementary material Fig. S1 and Movie 2). These data together with the RNAi experiment indicate that Eater is required cell-autonomously in individual plasmatocytes for attachment to the sessile compartment.

**Fig. 4.**
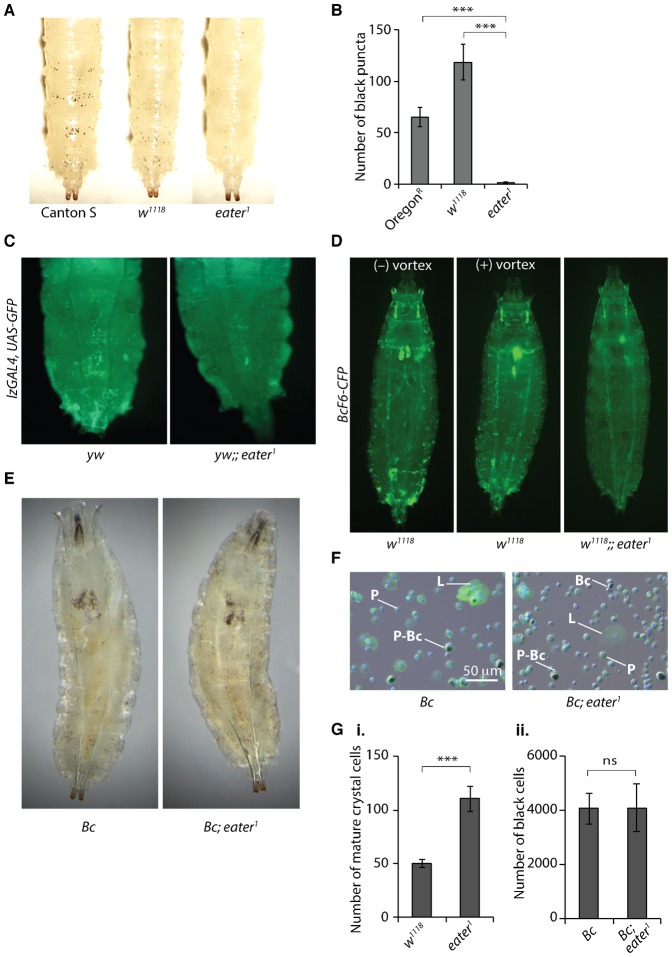
Sessile crystal cells are absent or almost absent in *eater^1^* mutant larvae. (A) Heating (67°C, 30 min) reveals the presence of superficial crystal cells in the posterior abdominal segments of Canton S, *w^1118^* and *eater^1^* null larvae. The ventral larval surface is shown. (B) Circumferential black puncta counts from the three posteriormost segments A6, A7 and A8 of heated *Oregon^R^*, *w^1118^* and *eater^1^* third instar larvae. (C) The crystal cell lineage marker *lzGAL4, UAS-GFP* reveals a near absence of sessile crystal cells in *eater^1^* third instar larvae compared to controls. Dorsal view of the 5 posterior-most abdominal segments. (D) The mature crystal cell marker *BcF6-CFP* reveals a near absence of sessile crystal cells in *eater^1^* mutant larvae compared to control *w^1118^* larvae. Vortexing (1 min, speed 10) is sufficient to release many sessile crystal cells. Genotypes: *w, EaterGAL4, UAS-2xeYFP; BcF6-CFP (P1+); msn9-mCherry* and *w, EaterGAL4, UAS-2xeYFP; BcF6-CFP (P1+); msn9-mCherry, eater^1^*. (E) The numbers of black cells in *Bc; eater^1^* mutant larvae are similar to the numbers in control larvae carrying the *Bc* gain-of-function mutation alone. Sessile black cells are present in *Bc* control larvae but absent in *Bc; eater^1^* larvae. (F) AF488-phalloidin and DAPI-stained hemocyte fields from *Bc* control and *Bc; eater^1^* third instar larvae. Plasmatocytes ‘P’ and lamellocytes ‘L’ are visible. Black cells may be anuclear black cells (Bc) or may coincide with plasmatocytes (P-Bc). Lamellocytes are a type of hemocyte induced by activation of the *Drosophila* cellular immune response. (G) (i) Cell counts of live crystal cells from hemocyte samples of *w^1118^* and *eater^1^* mutant larvae carrying the *BcF6-CFP* reporter transgene. Genotypes: *w, EaterGAL4, UAS-2xeYFP; BcF6-CFP (P1+); msn9-mCherry* and *w, EaterGAL4, UAS-2xeYFP; BcF6-CFP (P1+); msn9-mCherry, eater^1^*. (ii) Hemocytometry counts of black cells numbers from hemocyte samples of larvae carrying the *Bc* gain-of-function mutation either alone or in combination with the *eater^1^* mutation. Genotypes: *Bc* and *Bc; eater^1^*.

**Fig. 5.**
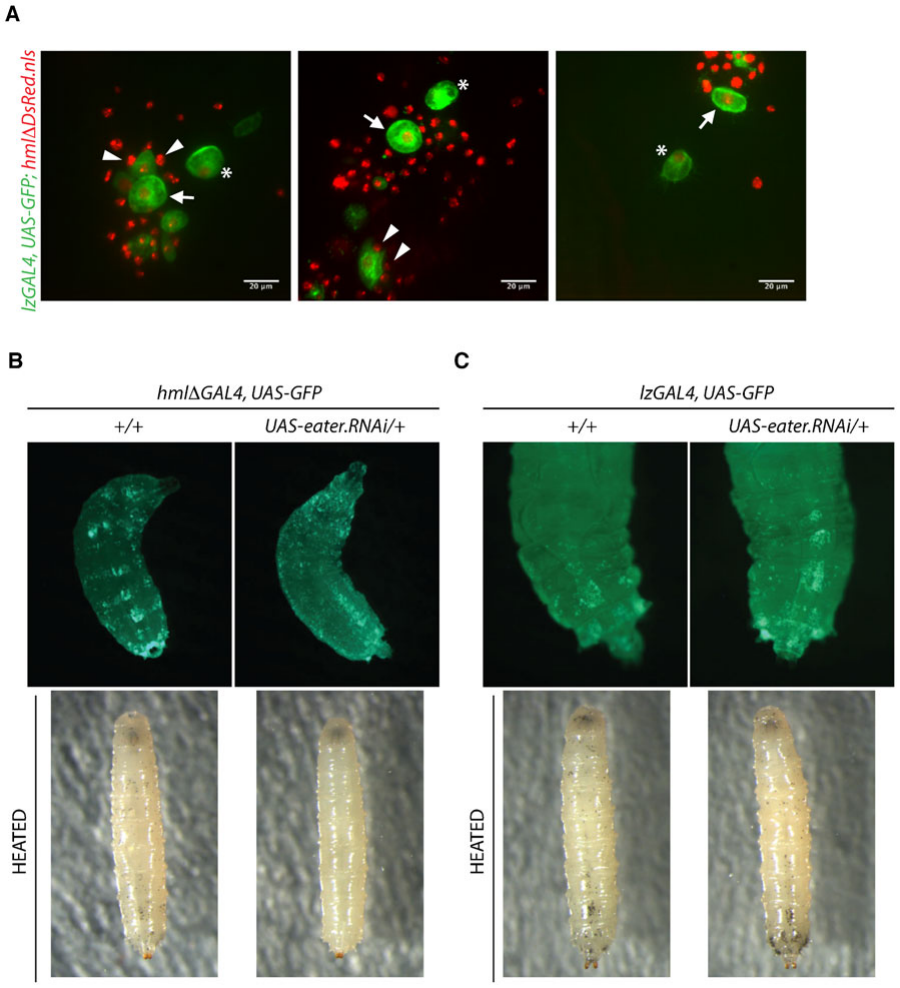
*eater* is required in plasmatocytes for sessile crystal cells. (A) Confocal images of dorsal sessile patches from larvae carrying the crystal cell marker *lzGAL4, UAS-GFP* and the plasmatocyte marker *HmlΔDsRed.nls*. Arrows indicate *lz>GFP* labeled sessile crystal cells associated with *HmlΔDsRed.nls* labeled sessile hemocytes. Arrowheads indicate tight associations or attachments between sessile crystal cells and nearest neighbor DsRed+ plasmatocytes in which the GFP labeled volume appears excluded by the unlabeled, cytoplasmic volume of a DsRed nuclear-labelled hemocyte. Asterisks indicate sessile crystal cells that appear to be plasmatocyte un-associated. Note, the *HmlΔDsRed.nls* transgene faintly labels the nuclei of a fraction of crystal cells expressing the *lzGAL4, UAS-GFP* marker. (B,C) Knockdown of *eater* in the *HmlΔ* hemocyte lineage but not the crystal cell (*Lz*) lineage causes an absence of sub-epidermal crystal cells. Panels at top, *gfp* images. Panels at bottom, bright-field images of heated larvae. (B) RNAi activity of the *UAS-eater.RNAi* transgene is confirmed by near absence of sessile plasmatocytes in *HmlΔ>gfp, eater.RNAi* larvae. Heating reveals a near absence of superficial (melanised) crystal cells in these larvae compared to controls. Genotypes: *w; HmlΔGAL4, UAS-GFP*, *w; HmlΔGAL4, UAS-GFP; UAS-eater.RNAi/+. *(C) *lz>gfp, eater.RNAi* larvae in which *eater* is knocked down in the crystal cell lineage still exhibit sessile crystal cells, visible both by *gfp* and by the heating assay. *yw, lzGAL4, UAS-GFP/w* and *yw, lzGAL4, UAS-GFP/w;; UAS-eater.RNAi/+*.

### Adherent *eater* deficient plasmatocytes are rounded and appear small on a glass surface

Eater localizes to the plasma membrane of *Drosophila* S2 cells (Chung and Kocks, 2011). We therefore hypothesized that the absence of attachment to the sessile compartment in *eater* deficient hemocytes could be due to a general requirement of Eater in hemocyte adhesion. A hallmark of impaired macrophage cell adhesion is a reduction in cell area on a substrate due to reduced cell spreading together with a decrease in the fraction of cells adhering (Fraser et al., 1993; Suzuki et al., 1997; Ribeiro et al., 2014). Spreading hemocytes on a cleaned glass slide, we observed that the cell areas of adherent *eater^1^* mutant hemocytes were small compared to those of control *w^1118^* hemocytes (Fig. 3D). We measured cell area and found that *w^1118^* adherent hemocytes have a mean cell area of 224 µm^2^ while *eater^1^* mutant adherent hemocytes have a mean cell area of 129 µm^2^ (Fig. 3E), consistent with a possible requirement of Eater in cell adhesion.

### Sessile crystal cells are absent or almost absent in *eater^1^* mutant larvae

We next asked whether *eater^1^* larvae also have defects in the other sessile hemocyte type, the crystal cell (Lanot et al., 2001). Like plasmatocytes, crystal cells may either be in a sessile or circulating state (Lanot et al., 2001). Heating larvae in water for 30 min at 67°C causes spontaneous activation of the pro-phenoloxidase zymogen within crystal cells and their subsequent blackening, making them visible through the cuticle as black puncta (Rizki et al., 1980). Surprisingly *eater^1^* larvae showed very few black puncta, unlike Canton^S^ and Oregon^R^ wild types and *w^1118^* controls (Fig. 4A,B). As heated larval tissues are opaque, the lack of black puncta in *eater^1^* mutant larvae could be due to an absence of crystal cells or to a reduction in the numbers of visible, sub-epidermal crystal cells (i.e. sessile cystal cells). We therefore combined the crystal cell lineage marker *lzGAL4, UAS-GFP* with the *eater^1^* mutation. Imaging whole larvae, we observed that sessile *Lz>GFP* labelled crystal cells were almost absent in *eater^1^* mutant larvae, compared to controls (Fig. 4C). We independently confirmed this result by combining *eater^1^* with the mature crystal cell fate marker *BcF6-CFP* (Gajewski et al., 2007) (Fig. 4D). While sessile crystal cells appeared absent, crystal cells were still visible in circulation in *eater^1^* mutant larvae (Fig. 4C,D).

To further examine the crystal cell population in *eater^1^* mutants, we combined the *eater^1^* mutation with *Black cells* (*Bc*), a mutation that causes blackening of crystal cells (Rizki et al., 1985; Lebestky et al., 2000; Lanot et al., 2001; Binggeli et al., 2014). The blackening of crystal cells in *Bc* homozygous larvae makes these cells readily visible through the cuticle as ‘black cells’ (Fig. 4E). Interestingly, though ‘black cells’ are dead crystal cells, they can still be sessile (*Bc* larva, Fig. 4E) and (Rizki et al., 1980). Observation of dissected hemocytes from the circulation of *Bc* control larvae revealed that melanised black cells often coincide with or have been ingested by plasmatocytes (Fig. 4F), as previously reported (Lanot et al., 2001; Gajewski et al., 2007). Examining larvae carrying both *Bc* and the *eater^1^* mutation revealed a near absence of sessile black cells while the total number of black cells appeared to be similar to that seen in *Bc* control larvae (Fig. 4E).

Previously, larval crystal cell numbers have been quantified by counting black puncta in heated larvae, as in Fig. 4A typically revealing between 40 and 100 crystal cells per larva (Lanot et al., 2001). Since *eater^1^* mutants lack sessile crystal cells, we used two alternative methods to count crystal cells. First, a fluorescence microscopy method based around counting live *BcF6-CFP* labeled crystal cells and second, a hemocytometry method based around counting black cells from larvae carrying the *Bc* mutation. Sessile hemocytes can be released by mechanical stimulation of the larval cuticle (Makhijani et al., 2011). In order to count both sessile and circulating crystal cells, larvae were vortexed for 1 min at max speed which releases sessile hemocytes (Petraki et al., 2015), including sessile crystal cells (Fig. 4D). Counting crystal cells using the *BcF6-CFP* label revealed that *eater^1^* mutant larvae had no lack of crystal cells; they appeared to have twice as many circulating crystal cells as *w^1118^* control larvae (Fig. 4Gi). Similarly, counting black cells numbers as a surrogate for mature crystal cell numbers, we found *Bc; eater^1^* mutant larvae had similar numbers of circulating black cells as *Bc* control larvae (Fig. 4Gii). Interestingly, it appears that the number of black cells in larvae carrying the *Bc* mutation is ∼4000, around 40–80 times more than the number of mature crystal cells in *w^1118^* larvae (Fig. 4Gi) or wild type larvae (Lanot et al., 2001).

These findings, that *eater^1^* mutant larvae lack all or almost all sessile crystal cells while retaining many crystal cells in circulation, suggests that the absence of *eater* does not impair crystal cell differentiation. Consistent with this, we observed that *eater^1^* crystal cells rupture on exposure to air like mature wild-type crystal cells (supplementary material Movies 3, 4).

### *eater* is required in plasmatocytes for sessile crystal cells

The near absence of sessile crystal cells was surprising as *eater* is expressed specifically in plasmatocytes but not in crystal cells (Kocks et al., 2005). We therefore asked how sessile crystal cells and sessile plasmatocytes are juxtaposed in the sessile compartment in third instar larvae. To do this, we used double live confocal imaging of the *LzGAL4, UAS-GFP* marker, which only labels crystal cells and the *HmlΔDsRed.nls* marker, which labels plasmatocytes but not mature crystal cells. Imaging revealed that most sessile GFP+ crystal cells are closely associated with sessile DsRed+ hemocytes (Fig. 5A). It is interesting to note that crystal cell and plasmatocyte tight associations are also frequently observed in hemolymph preparations from either wild-type or *eater^1^* mutant larvae (supplementary material Movies 3, 4).

Following from our observation that sessile crystal cells are almost absent in *eater^1^* mutant larvae, we asked whether lack of sessile crystal cells in *eater^1^* larvae is due to a requirement of *eater* in the crystal cell *Lz* lineage or the *Hml* lineage. To decipher between these two possibilities, we knocked down *eater* in the plasmatocyte lineage using *HmlΔGAL4* driver or in the crystal cell lineage using *lzGAl4* and used the heating assay to monitor the presence of sessile crystal cells. Interestingly, *lzGAL4>eater-RNAi* larvae still exhibited sub-epidermal crystal cells like wild type larvae, as observed by heating and black puncta visualization or by GFP fluorescence (Fig. 5C). However, sub-epidermal crystal cells were absent from *HmlΔGAL4>eater-RNAi* larvae, as viewed by heating and black puncta visualization (Fig. 5B). Thus, crystal cell sessility appears to require Eater non cell-autonomously in hemocytes of the *Hml+* lineage.

## DISCUSSION

The transmembrane receptor Eater was initially described as a major phagocytic receptor, recognizing a broad range of bacteria or bacterial products (Kocks et al., 2005). This conclusion originated from *ex-vivo* analysis of phagocytosis in S2 cells, using RNAi knockdown of *eater*, and hemocytes from larvae ablated for *eater* by overlapping deficiencies. Although our study confirms that Eater contributes to the phagocytosis of Gram-positive bacteria, it does not reveal any role of Eater in the phagocytosis of Gram-negative bacteria. Thus, the use of a clean deletion of *eater* demonstrates that the phagocytic activity of this receptor as measured by *ex vivo* analysis is more restricted than expected. At this stage, we cannot exclude the possibility that Eater contributes to phagocytosis of Gram-negative bacteria *in vivo*, where secreted factors (eg. opsonins) could assist Eater-mediated phagocytosis in the hemolymph. The very different surface chemistries of Gram-positive and Gram-negative bacteria (Mengin-Lecreulx and Lemaitre, 2005; Lemaitre and Hoffmann, 2007), namely peptidoglycan-based cell wall versus lipopolysaccharide-based outer membrane, respectively, could explain the differential requirement of Eater in Gram-positive but not Gram-negative uptake. The results obtained with the *eater* mutant imply different phagocytic mechanisms for uptake of Gram-positive and Gram-negative bacteria. The phagocytosis of Gram-positive bacteria in *Drosophila* also requires the cell surface receptors Draper and Integrin βν (Hashimoto et al., 2009; Ulvila et al., 2011; Shiratsuchi et al., 2012). Whether Eater interacts with these proteins and how it contributes to phagocytosis remain an open question.

Our data suggest that *eater* deficient larvae do not have a gross defect in plasmatocyte maturation or differentiation. Indeed, *eater* deficient plasmatocytes are competent to phagocytose Gram-negative bacteria and express the mature plasmatocyte-specific marker NimC1 like wild-type hemocytes (Fig. 3D). Importantly, we have discovered a cell-autonomous requirement of *eater* in plasmatocytes for their association to the sessile compartment. To our knowledge, the *eater* deletion is the first loss-of-function mutation identified in *Drosophila* that causes an absence or a near absence of the sessile hemocyte state. Our RNAi and MARCM mosaic analysis confirm that *eater* is required specifically in individual plasmatocyte for sessility and not in another cell type, consistent with expression data (Kocks et al., 2005). We therefore suggest that the Eater receptor functions in some way to enable hemocyte adhesion at the sessile compartment. Our study does not distinguish whether Eater is required to promote hemocyte migration to the sessile compartment or maintain hemocytes at the sessile compartment. How a receptor such as Eater can contribute to two distinct functions, phagocytosis and sessility, is intriguing. The observation that *eater* deficient plasmatocytes fail to spread as much as the wild type on a glass slide suggests Eater could act as a cell adhesion receptor. A function in cell adhesion could explain why *eater* deficient hemocytes do not remain attached to the sessile niche as well as the defects observed in phagocytosis of Gram-positive bacteria. In the same line, mutation in the *Drosophila* integrin βν receptor also cause multiple defects in both phagocytosis and encapsulation (Howell et al., 2012; Shiratsuchi et al., 2012).

In *eater^1^* mutant larvae, both plasmatocytes and crystal cells appear to be absent or almost absent from the sessile compartment. We have shown that sessile crystal cells require Eater in *Hml+* plasmatocytes. This indicates that absence of sessile crystal cells in the *eater* mutant is a secondary consequence of the absence of sessile plasmatocytes. A first explanation is that crystal cells attach to sessile plasmatocytes to adhere to the sessile compartment. A second hypothesis is that sessile crystal cells derive from sessile hemocytes of the *Hml+* lineage. The latter hypothesis is supported by observation that *Lz-GAL4, UAS-GFP* sessile crystal cells derive from *HmlΔ.dsRed.nls* expressing sessile hemocytes (Leitao and Sucena, personal communication 2015). This is also consistent with recent observations showing that *Drosophila* hematopoiesis is not restricted to the embryonic stage or to the lymph gland but takes place in the circulation and in the sessile compartment with higher plasticity between hemocyte lineages than first thought (Lanot et al., 2001; Márkus et al., 2009; Avet-Rochex et al., 2010; Honti et al., 2010). The absence of sessile hemocytes in *eater^1^* null larvae provide a unique tool to assess the function of the sessile compartment in the context of development, peripheral nervous system stimulation (Makhijani et al., 2011) or immune challenge (Márkus et al., 2009).

We have not addressed the ligand of the Eater receptor in this study. However, it is interesting to note that in absence of *eater*, essentially all plasmatocytes are absent from the sessile state. This suggests that all plasmatocytes use the same form of attachment site to attach to both lateral and dorsal patches of the sessile compartment. Clues as to what may form a hemocyte attachment site come from anatomy: invertebrate epithelial cells form junctions to the apical extracellular matrix (ECM) lining the larval cuticle and to the ECM lining the basal surface (Brown, 2011). Interestingly, electron microscopic cross sections through sessile plasmatocytes show attachment to the basal surface of cuticular epithelial cells (Lanot et al., 2001). Additionally, hemocytes appear to reside in the sessile compartment in close proximity to the endings of peripheral neurons and presumably their associated glial cells (Makhijani et al., 2011). Sessile hemocytes appear to cluster around oenocytes, secretory cells, which synthesize and secrete hydrocarbons onto the larval cuticle and may also contribute to endocrine regulation (Makki et al., 2014). Whether sessile hemocytes attach to a component of the ECM or directly to different cell types within the epithelial wall, remains to be determined.

Although the Nimrod gene family is thought to be an important component of insect innate host defense, few members of this family have been the focus of functional studies (Somogyi et al., 2008). The best characterized Nimrod gene is Draper, an atypical family member having only 1 Nim repeat and 15 EGF-like repeats, which is expressed in glia, hemocytes and other tissues and mediates engulfment of apoptotic cells during development and phagocytosis of bacteria during infection (Manaka et al., 2004; Awasaki et al., 2006; Shiratsuchi et al., 2012). An *in vivo RNAi* study has pointed to a role of NimC1 in the phagocytosis of the Gram-positive bacteria *S. aureus* (Kurucz et al., 2007). Here, we uncover a new role for Eater protein in hemocytes adhesion and localization. This raises the hypothesis that other Nimrod family members could also play a role in hemocyte homing by modulating their adhesion properties. Future genetic study should address the function of other Nimrod family member to better decipher the role of this family.

## Supplementary Material

Supplementary Material

## References

[b1] Alberts B. Johnson A. Lewis J. Raff M. Roberts K. Walter P. (2002). Molecular Biology of the Cell

[b2] Avet-Rochex A. Boyer K. Polesello C. Gobert V. Osman D. Roch F. Augé B. Zanet J. Haenlin M. Waltzer L. (2010). An in vivo RNA interference screen identifies gene networks controlling Drosophila melanogaster blood cell homeostasis. BMC Dev. Biol. 10, 65 10.1186/1471-213X-10-65 20540764 PMC2891661

[b3] Awasaki T. Tatsumi R. Takahashi K. Arai K. Nakanishi Y. Ueda R. Ito K. (2006). Essential role of the apoptotic cell engulfment genes draper and ced-6 in programmed axon pruning during Drosophila metamorphosis. Neuron 50, 855–867 10.1016/j.neuron.2006.04.027 16772168

[b4] Babcock D. T. Brock A. R. Fish G. S. Wang Y. Perrin L. Krasnow M. A. Galko M. J. (2008). Circulating blood cells function as a surveillance system for damaged tissue in Drosophila larvae. Proc. Natl. Acad. Sci. USA 105, 10017–10022 10.1073/pnas.0709951105 18632567 PMC2474562

[b5] Baena-Lopez L. A. Alexandre C. Mitchell A. Pasakarnis L. Vincent J. P. (2013). Accelerated homologous recombination and subsequent genome modification in Drosophila. Development 140, 4818–4825 10.1242/dev.100933 24154526 PMC3833436

[b6] Binggeli O. Neyen C. Poidevin M. Lemaitre B. (2014). Prophenoloxidase activation is required for survival to microbial infections in Drosophila. PLoS Pathog. 10, e1004067 10.1371/journal.ppat.1004067 24788090 PMC4006879

[b7] Brown N. H. (2011). Extracellular matrix in development: insights from mechanisms conserved between invertebrates and vertebrates. Cold Spring Harb. Perspect. Biol. 3, a005082 10.1101/cshperspect.a005082 21917993 PMC3225944

[b8] Charroux B. Royet J. (2009). Elimination of plasmatocytes by targeted apoptosis reveals their role in multiple aspects of the Drosophila immune response. Proc. Natl. Acad. Sci. USA 106, 9797–9802 10.1073/pnas.0903971106 19482944 PMC2700997

[b9] Chung Y. S. Kocks C. (2011). Recognition of pathogenic microbes by the Drosophila phagocytic pattern recognition receptor Eater. J. Biol. Chem. 286, 26524–26532 10.1074/jbc.M110.214007 21613218 PMC3143617

[b10] Crozatier M. Meister M. (2007). Drosophila haematopoiesis. Cell. Microbiol. 9, 1117–1126 10.1111/j.1462-5822.2007.00930.x 17394559

[b11] Defaye A. Evans I. Crozatier M. Wood W. Lemaitre B. Leulier F. (2009). Genetic ablation of Drosophila phagocytes reveals their contribution to both development and resistance to bacterial infection. J. Innate Immun. 1, 322–334 10.1159/000210264 20375589

[b12] Evans C. J. Banerjee U. (2003). Transcriptional regulation of hematopoiesis in Drosophila. Blood Cells Mol. Dis. 30, 223–228 10.1016/S1079-9796(03)00028-7 12732186

[b13] Evans C. J. Liu T. Banerjee U. (2014). Drosophila hematopoiesis: markers and methods for molecular genetic analysis. Methods 68, 242–251 10.1016/j.ymeth.2014.02.038 24613936 PMC4051208

[b14] Fox J. (2005). Getting started with the R commander: a basic-statistics graphical user interface to R. *J. Stat.* Softw. 14, 1–42.

[b15] Fraser I. Hughes D. Gordon S. (1993). Divalent cation-independent macrophage adhesion inhibited by monoclonal antibody to murine scavenger receptor. Nature 364, 343–346 10.1038/364343a0 8332192

[b16] Gajewski K. M. Sorrentino R. P. Lee J. H. Zhang Q. Russell M. Schulz R. A. (2007). Identification of a crystal cell-specific enhancer of the black cells prophenoloxidase gene in Drosophila. Genesis 45, 200–207 10.1002/dvg.20285 17417793

[b17] Hashimoto Y. Tabuchi Y. Sakurai K. Kutsuna M. Kurokawa K. Awasaki T. Sekimizu K. Nakanishi Y. Shiratsuchi A. (2009). Identification of lipoteichoic acid as a ligand for draper in the phagocytosis of Staphylococcus aureus by Drosophila hemocytes. J. Immunol. 183, 7451–7460 10.4049/jimmunol.0901032 19890048

[b18] Honti V. Csordás G. Márkus R. Kurucz E. Jankovics F. Andó I. (2010). Cell lineage tracing reveals the plasticity of the hemocyte lineages and of the hematopoietic compartments in Drosophila melanogaster. Mol. Immunol. 47, 1997–2004 10.1016/j.molimm.2010.04.017 20483458

[b19] Honti V. Csordás G. Kurucz É. Márkus R. Andó I. (2014). The cell-mediated immunity of Drosophila melanogaster: hemocyte lineages, immune compartments, microanatomy and regulation. Dev. Comp. Immunol. 42, 47–56 10.1016/j.dci.2013.06.005 23800719

[b20] Howell L. Sampson C. J. Xavier M. J. Bolukbasi E. Heck M. M. Williams M. J. (2012). A directed miniscreen for genes involved in the Drosophila anti-parasitoid immune response. Immunogenetics 64, 155–161 10.1007/s00251-011-0571-3 21947570

[b21] Jung S. H. Evans C. J. Uemura C. Banerjee U. (2005). The Drosophila lymph gland as a developmental model of hematopoiesis. Development 132, 2521–2533 10.1242/dev.01837 15857916

[b22] Kocks C. Cho J. H. Nehme N. Ulvila J. Pearson A. M. Meister M. Strom C. Conto S. L. Hetru C. Stuart L. M. (2005). Eater, a transmembrane protein mediating phagocytosis of bacterial pathogens in Drosophila. Cell 123, 335–346 10.1016/j.cell.2005.08.034 16239149

[b23] Kurucz E. Zettervall C. J. Sinka R. Vilmos P. Pivarcsi A. Ekengren S. Hegedüs Z. Ando I. Hultmark D. (2003). Hemese, a hemocyte-specific transmembrane protein, affects the cellular immune response in Drosophila. Proc. Natl. Acad. Sci. USA 100, 2622–2627 10.1073/pnas.0436940100 12598653 PMC151390

[b24] Kurucz E. Márkus R. Zsámboki J. Folkl-Medzihradszky K. Darula Z. Vilmos P. Udvardy A. Krausz I. Lukacsovich T. Gateff E. (2007). Nimrod, a putative phagocytosis receptor with EGF repeats in Drosophila plasmatocytes. Curr. Biol. 17, 649–654 10.1016/j.cub.2007.02.041 17363253

[b25] Lanot R. Zachary D. Holder F. Meister M. (2001). Postembryonic hematopoiesis in Drosophila. Dev. Biol. 230, 243–257 10.1006/dbio.2000.0123 11161576

[b26] Lebestky T. Chang T. Hartenstein V. Banerjee U. (2000). Specification of Drosophila hematopoietic lineage by conserved transcription factors. Science 288, 146–149 10.1126/science.288.5463.146 10753120

[b27] Lee T. Luo L. (1999). Mosaic analysis with a repressible cell marker for studies of gene function in neuronal morphogenesis. Neuron 22, 451–461 10.1016/S0896-6273(00)80701-1 10197526

[b200] Leitão A. B. Sucena E. (2015). Drosophila sessile hemocyte clusters are true hematopoietic tissues that regulate larval blood cell differentiation Elife in press. 10.7554/eLife.06166 PMC4357286 25650737

[b28] Lemaitre B. Hoffmann J. (2007). The host defense of Drosophila melanogaster. Annu. Rev. Immunol. 25, 697–743 10.1146/annurev.immunol.25.022106.141615 17201680

[b29] Makhijani K. Alexander B. Tanaka T. Rulifson E. Brückner K. (2011). The peripheral nervous system supports blood cell homing and survival in the Drosophila larva. Development 138, 5379–5391 10.1242/dev.067322 22071105 PMC3222213

[b201] Makhijani K. Brückner K. (2012). Of blood cells and the nervous system: hematopoiesis in the Drosophila larva. Fly (Austin). 6, 254–60. 23022764 10.4161/fly.22267 PMC3519660

[b30] Makki R. Cinnamon E. Gould A. P. (2014). The development and functions of oenocytes. Annu. Rev. Entomol. 59, 405–425 10.1146/annurev-ento-011613-162056 24397521 PMC7613053

[b31] Manaka J. Kuraishi T. Shiratsuchi A. Nakai Y. Higashida H. Henson P. Nakanishi Y. (2004). Draper-mediated and phosphatidylserine-independent phagocytosis of apoptotic cells by Drosophila hemocytes/macrophages. J. Biol. Chem. 279, 48466–48476 10.1074/jbc.M408597200 15342648

[b32] Márkus R. Laurinyecz B. Kurucz E. Honti V. Bajusz I. Sipos B. Somogyi K. Kronhamn J. Hultmark D. Andó I. (2009). Sessile hemocytes as a hematopoietic compartment in Drosophila melanogaster. Proc. Natl. Acad. Sci. USA 106, 4805–4809 10.1073/pnas.0801766106 19261847 PMC2660760

[b33] Mengin-Lecreulx D. Lemaitre B. (2005). Structure and metabolism of peptidoglycan and molecular requirements allowing its detection by the Drosophila innate immune system. J. Endotoxin Res. 11, 105–111 10.1179/096805105X35233 15949137

[b34] Minakhina S. Druzhinina M. Steward R. (2007). Zfrp8, the Drosophila ortholog of PDCD2, functions in lymph gland development and controls cell proliferation. Development 134, 2387–2396 10.1242/dev.003616 17522156

[b35] Neyen C. Bretscher A. J. Binggeli O. Lemaitre B. (2014). Methods to study Drosophila immunity. Methods 68, 116–128 10.1016/j.ymeth.2014.02.023 24631888

[b300] Petraki S. Alexander B. Bruückner K. (2015). Assaying blood cell populations of the Drosophila melanogaster larva JoVE in press 10.3791/52733 PMC4692709 26650404

[b36] **R Development Core Team**(2008). R: A Language and Environment for Statistical Computing R Foundation for Statistical Computing, Vienna, Austria. Available at http://www.R-project.org/

[b37] Ribeiro S. A. D'Ambrosio M. V. Vale R. D. (2014). Induction of focal adhesions and motility in Drosophila S2 cells. Mol. Biol. Cell 25, 3861–3869 10.1091/mbc.E14-04-0863 25273555 PMC4244196

[b38] Rizki T. M. Rizki R. M. (1992). Lamellocyte differentiation in Drosophila larvae parasitized by Leptopilina. Dev. Comp. Immunol. 16, 103–110 10.1016/0145-305X(92)90011-Z 1499832

[b39] Rizki T. M. Rizki R. M. Grell E. (1980). A mutant affecting the crystal cells in Drosophila melanogaster. Roux's Arch. Dev. Biol. 188, 91–99 10.1007/BF00848799 28304971

[b40] Rizki T. M. Rizki R. M. Bellotti R. A. (1985). Genetics of a Drosophila phenoloxidase. Mol. Gen. Genet. 201, 7–13 10.1007/BF00397978 3932822

[b41] Shiratsuchi A. Mori T. Sakurai K. Nagaosa K. Sekimizu K. Lee B. L. Nakanishi Y. (2012). Independent recognition of Staphylococcus aureus by two receptors for phagocytosis in Drosophila. J. Biol. Chem. 287, 21663–21672 10.1074/jbc.M111.333807 22547074 PMC3381130

[b42] Sinenko S. A. Kim E. K. Wynn R. Manfruelli P. Ando I. Wharton K. A. Perrimon N. Mathey-Prevot B. (2004). Yantar, a conserved arginine-rich protein is involved in Drosophila hemocyte development. Dev. Biol. 273, 48–62 10.1016/j.ydbio.2004.05.022 15302597

[b43] Somogyi K. Sipos B. Pénzes Z. Kurucz E. Zsámboki J. Hultmark D. Andó I. (2008). Evolution of genes and repeats in the Nimrod superfamily. Mol. Biol. Evol. 25, 2337–2347 10.1093/molbev/msn180 18703524

[b44] Suzuki H. Kurihara Y. Takeya M. Kamada N. Kataoka M. Jishage K. Ueda O. Sakaguchi H. Higashi T. Suzuki T. (1997). A role for macrophage scavenger receptors in atherosclerosis and susceptibility to infection. Nature 386, 292–296 10.1038/386292a0 9069289

[b45] Ulvila J. Vanha-Aho L. M. Rämet M. (2011). Drosophila phagocytosis - still many unknowns under the surface. APMIS 119, 651–662 10.1111/j.1600-0463.2011.02792.x 21917002

[b46] Watson F. L. Püttmann-Holgado R. Thomas F. Lamar D. L. Hughes M. Kondo M. Rebel V. I. Schmucker D. (2005). Extensive diversity of Ig-superfamily proteins in the immune system of insects. Science 309, 1874–1878 10.1126/science.1116887 16109846

[b47] Welman A. Serrels A. Brunton V. G. Ditzel M. Frame M. C. (2010). Two-color photoactivatable probe for selective tracking of proteins and cells. J. Biol. Chem. 285, 11607–11616 10.1074/jbc.M110.102392 20139076 PMC2857038

[b48] Zettervall C. J. Anderl I. Williams M. J. Palmer R. Kurucz E. Ando I. Hultmark D. (2004). A directed screen for genes involved in Drosophila blood cell activation. Proc. Natl. Acad. Sci. USA 101, 14192–14197 10.1073/pnas.0403789101 15381778 PMC521135

