# Supplementary material for: The Nimrod transmembrane receptor Eater is required for hemocyte attachment to the sessile compartment in Drosophila melanogaster
Source: Biol Open. 2015 Feb 13;4(3):355–63. doi: 10.1242/bio.201410595 (PMC4359741; doi:10.1242/bio.201410595)
Supplement: Supplementary Material [file supp_4_3_355__index.html]

The Nimrod transmembrane receptor Eater is required for hemocyte attachment to the sessile compartment in Drosophila melanogaster — Supplementary Material 

# The Nimrod transmembrane receptor Eater is required for hemocyte attachment to the sessile compartment in *Drosophila melanogaster*

## bio.201410595 Supplementary Material

**Files in this Data Supplement:**

- Supplementary Material - Andrew J. Bretscher et al. doi: 10.1242/bio.201410595
- Movie 1 - ***eater* is required cell-autonomously in hemocytes for their binding to the sessile compartment.** Wild type MARCM clones of *gfp+* hemocytes, dorsally located. The majority of sessile *gfp+* hemocytes remain stationary with respect to the cuticle as the larva moves. Note, as a result of the MARCM technique, non-hemocyte somatic *gfp+* clones are also present in mosaic larvae. Genotype of MARCM clone: *y, hsFLP, ptubGAL4, UAS-GFP/(w/Y);;; FRT82B/FRT82B*.
- Movie 2 - ***eater* is required cell-autonomously in hemocytes for their binding to the sessile compartment.** (See legend of Movie 1). Mutant *eater1* clones of *gfp+* hemocytes, dorsally located. The majority of *gfp+* hemocytes do not remain stationary with respect to the larval cuticle as the larva moves. The 2-cell (non-hemocyte) somatic clone at the top of the movie frame, and indicated in supplementary material Fig. S1B by a pair of red arrowheads, provides an internal reference for movement within the frame. Genotype of MARCM clone: *y, hsFLP, ptubGAL4, UAS-GFP/(w/Y);; FRT82B, eater1/FRT82B, eater1*.
- Movie 3 - **Dissected, live mount wild type and *eater1* mature crystal cells usually rupture within 2 min of exposure to air, further suggesting that crystal cell maturation is intact in *eater1* mutant larvae.** Wild type hemocytes. Tight associations are seen between plasmatocytes or plasmatocyte-like hemocytes and crystal cells, both from control and *eater1* mutant larvae. Genotypes: *w1118, EaterGAL4, UAS-2xeYFP; BcF6-CFP (P1+); msn9-mCherry* and *w, EaterGAL4, UAS-2xeYFP; BcF6-CFP (P1+); msn9-mCherry, eater1*.
- Movie 4 - **Dissected, live mount wild type and *eater*1 mature crystal cells usually rupture within 2 min of exposure to air, further suggesting that crystal cell maturation is intact in *eater*1 mutant larvae.** (See legend of Movie 3). *eater1* mutant hemocytes.
