# Supplementary material for: The Nimrod transmembrane receptor Eater is required for hemocyte attachment to the sessile compartment in Drosophila melanogaster
Source: Biol Open. 2015 Feb 13;4(3):355–63. doi: 10.1242/bio.201410595 (PMC4359741; doi:10.1242/bio.201410595)
Supplement: Supplementary Material [file supp_bio.201410595_bio.201410595-s1.pdf]

Biology Open (2015) 000, 1–9 doi:10.1242/bio.201410595

Supplementary Material

Andrew J. Bretscher et al. doi: 10.1242/bio.201410595

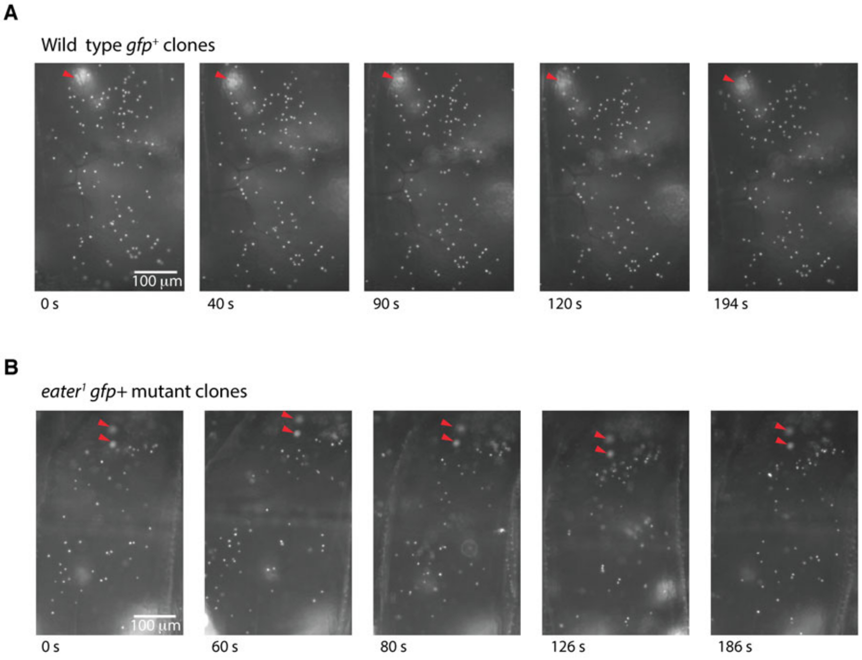

Fig. S1. ***eater*** **is required cell-autonomously in hemocytes for their binding to the sessile compartment.** (A) Still images of dorsal wild type *gfp+* hemocyte clones across a 194s interval. Images are punctuated by larval movement. An out-of-focus non-hemocyte *gfp+* clone is marked by a red arrowhead and, together with segment boundaries, serves as an internal tissue reference for movement within the frame. Data from supplementary material Movie 1. Genotype of MARCM clones: *y, hsFLP, ptubGAL4, UAS-GFP/(w/Y);; FRT82B/FRT82B*. Genotype of larva: *y, hsFLP, ptubGAL4, UAS-GFP/(w/Y);; FRT82B/FRT82B, ptubGAL80*. (B) Dorsal view of $eater^1$ mutant *gfp+* hemocyte clones across a 186s interval. Images are punctuated by larval movement. Two out-of-focus non-hemocyte *gfp+* cells are marked by a pair of red arrowheads and, together with segment boundaries, serve as an internal tissue reference for movement within the frame. Data from supplementary material Movie 2. Genotype of MARCM clones: *y, hsFLP, ptubGAL4, UAS-GFP/(w/Y);; FRT82B,* $eater^1$*/FRT82B,* $eater^1$. Genotype of larva: *y, hsFLP, ptubGAL4, UAS-GFP/(w/Y);; FRT82B,* $eater^1$*/FRT82B, ptubGAL80*.

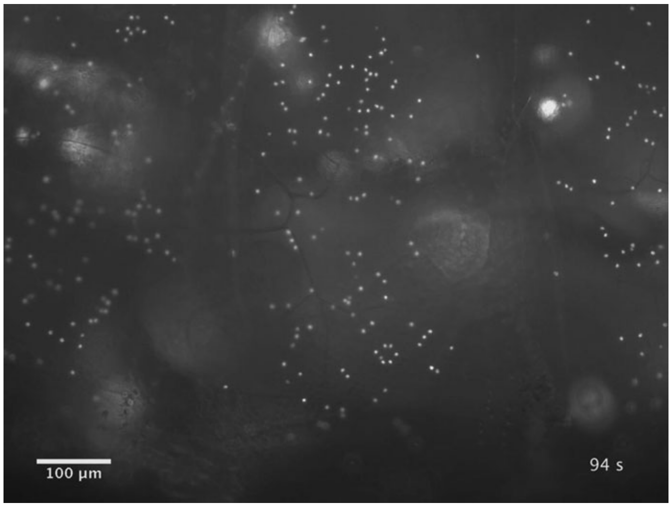

Movie 1. ***eater*** **is required cell-autonomously in hemocytes for their binding to the sessile compartment.** Wild type MARCM clones of *gfp+* hemocytes, dorsally located. The majority of sessile *gfp+* hemocytes remain stationary with respect to the cuticle as the larva moves. Note, as a result of the MARCM technique, non-hemocyte somatic *gfp+* clones are also present in mosaic larvae. Genotype of MARCM clone: *y, hsFLP, ptubGAL4, UAS-GFP/(w/Y);; FRT82B/FRT82B*.

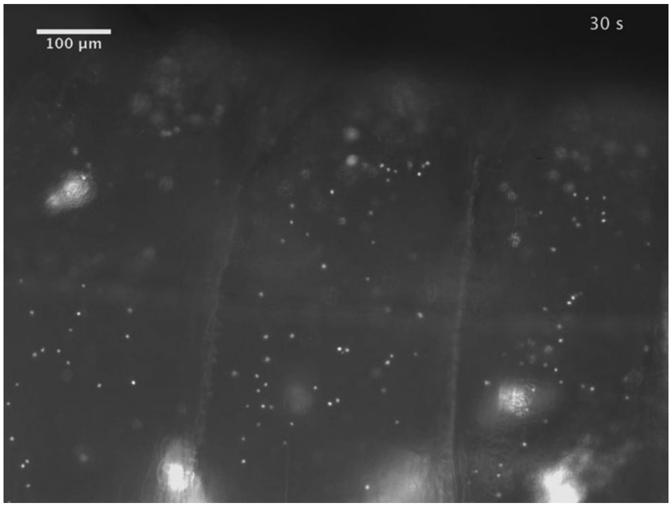

Movie 2. ***eater*** **is required cell-autonomously in hemocytes for their binding to the sessile compartment.** (See legend of Movie 1). Mutant $eater^1$ clones of *gfp+* hemocytes, dorsally located. The majority of *gfp+* hemocytes do not remain stationary with respect to the larval cuticle as the larva moves. The 2-cell (non-hemocyte) somatic clone at the top of the movie frame, and indicated in supplementary material Fig. S1B by a pair of red arrowheads, provides an internal reference for movement within the frame. Genotype of MARCM clone: *y, hsFLP, ptubGAL4, UAS-GFP/(w/Y);; FRT82B,* $eater^1$*/FRT82B,* $eater^1$.

Biology Open

Biology Open (2015) 000, 1–9 doi:10.1242/bio.201410595

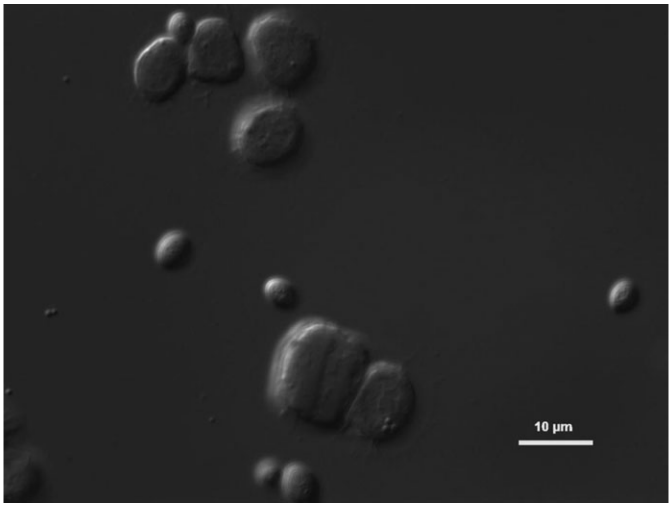

Movie 3. Dissected, live mount wild type and *eater*^{1} mature crystal cells usually rupture within 2 min of exposure to air, further suggesting that crystal cell maturation is intact in *eater*^{1} mutant larvae. Wild type hemocytes. Tight associations are seen between plasmatocytes or plasmatocyte-like hemocytes and crystal cells, both from control and *eater*^{1} mutant larvae. Genotypes: *w*^{1118}*, EaterGAL4, UAS-2xeYFP; BcF6-CFP (P1*$^+$*); msn9-mCherry* and *w, EaterGAL4, UAS-2xeYFP; BcF6-CFP (P1*$^+$*); msn9-mCherry, eater*^{1}.

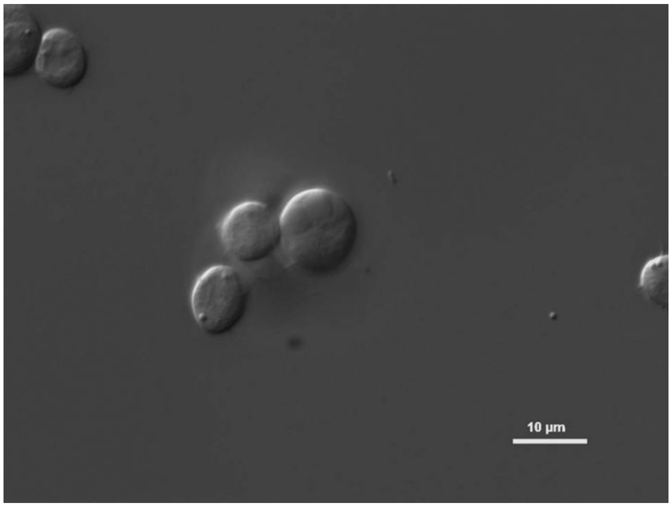

Movie 4. Dissected, live mount wild type and *eater*^{1} mature crystal cells usually rupture within 2 min of exposure to air, further suggesting that crystal cell maturation is intact in *eater*^{1} mutant larvae. (See legend of Movie 3). *eater*^{1} mutant hemocytes.

Biology Open
